# Hotel booking demand datasets

**DOI:** 10.1016/j.dib.2018.11.126

**Published:** 2018-11-29

**Authors:** Nuno Antonio, Ana de Almeida, Luis Nunes

**Affiliations:** a Instituto Universitário de Lisboa (ISCTE-IUL), Lisbon, Portugal; b Instituto de Telecomunicações, Lisbon, Portugal; c CISUC, Coimbra, Portugal; d ISTAR-IUL, Lisbon, Portugal

## Abstract

This data article describes two datasets with hotel demand data. One of the hotels (H1) is a resort hotel and the other is a city hotel (H2). Both datasets share the same structure, with 31 variables describing the 40,060 observations of H1 and 79,330 observations of H2. Each observation represents a hotel booking. Both datasets comprehend bookings due to arrive between the 1st of July of 2015 and the 31st of August 2017, including bookings that effectively arrived and bookings that were canceled. Since this is hotel real data, all data elements pertaining hotel or costumer identification were deleted. Due to the scarcity of real business data for scientific and educational purposes, these datasets can have an important role for research and education in revenue management, machine learning, or data mining, as well as in other fields.

**Specifications table**

**Table:** 

Subject area	Hospitality Management
More specific subject area	Revenue Management
Type of data	Text files and R objects
How data was acquired	Extraction from hotels’ Property Management System (PMS) SQL databases
Data format	Mixed (raw and preprocessed)
Experimental factors	Some of the variables were engineered from other variables from different database tables. The data point time for each observation was defined as the day prior to each booking׳s arrival
Experimental features	Data was extracted via TSQL queries executed directly in the hotels’ PMS databases and R was employed to perform data analysis
Data source location	Both hotels are located in Portugal: H1 at the resort region of Algarve and H2 at the city of Lisbon
Data accessibility	Data is supplied with the paper

**Value of the data**

• Descriptive analytics can be employed to further understand patterns, trends, and anomalies in data;
• Used to perform research in different problems like: bookings cancellation prediction, customer segmentation, customer satiation, seasonality, among others;
• Researchers can use the datasets to benchmark bookings’ prediction cancellation models against results already known (e.g. [1]);
• Machine learning researchers can use the datasets for benchmarking the performance of different algorithms for solving the same type of problem (classification, segmentation, or other);
• Educators can use the datasets for machine learning classification or segmentation problems;
• Educators can use the datasets to obtain either statistics or data mining training.

## 1 Data

In tourism and travel related industries, most of the research on Revenue Management demand forecasting and prediction problems employ data from the aviation industry, in the format known as the Passenger Name Record (PNR). This is a format developed by the aviation industry [2]. However, the remaining tourism and travel industries like hospitality, cruising, theme parks, etc., have different requirements and particularities that cannot be fully explored without industry׳s specific data. Hence, two hotel datasets with demand data are shared to help in overcoming this limitation.

The datasets now made available were collected aiming at the development of prediction models to classify a hotel booking׳s likelihood to be canceled. Nevertheless, due to the characteristics of the variables included in these datasets, their use goes beyond this cancellation prediction problem.

One of the most important properties in data for prediction models is not to promote leakage of future information [3]. In order to prevent this from happening, the timestamp of the target variable must occur after the input variables’ timestamp. Thus, instead of directly extracting variables from the bookings database table, when available, the variables’ values were extracted from the bookings change log, with a timestamp relative to the day prior to arrival date (for all the bookings created before their arrival date).

**Fig. 1:**
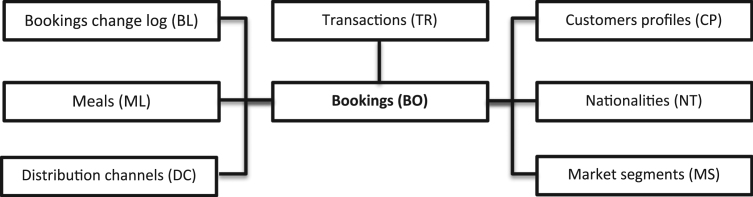
Diagram of PMS database tables where variables where extracted from.

Not all variables in these datasets come from the bookings or change log database tables. Some come from other tables, and some are engineered from different variables from different tables. A diagram presenting the PMS database tables from where variables were extracted is presented in Fig. 1. A detailed description of each variable is offered in the following section.

## 2 Experimental design, materials and methods

Data was obtained directly from the hotels’ PMS databases’ servers by executing a TSQL query on SQL Server Studio Manager, the integrated environment tool for managing Microsoft SQL databases [4]. This query first collected the value or ID (in the case of foreign keys) of each variable in the BO table. The BL table was then checked for any alteration with respect to the day prior to the arrival. If an alteration was found, the value used was the one present in the BL table. For all the variables holding values in related tables (like meals, distribution channels, nationalities or market segments), their related values were retrieved. A detailed description of the extracted variables, their origin, and the engineering procedures employed in its creation is shown in Table 1.

**Table 1:** Variables description.

**Variable**	**Type**	**Description**	**Source/Engineering**
*ADR*	Numeric	Average Daily Rate as defined by [5]	BO, BL and TR / Calculated by dividing the sum of all lodging transactions by the total number of staying nights
*Adults*	Integer	Number of adults	BO and BL
*Agent*	Categorical	ID of the travel agency that made the booking^a^	BO and BL
*ArrivalDateDayOfMonth*	Integer	Day of the month of the arrival date	BO and BL
*ArrivalDateMonth*	Categorical	Month of arrival date with 12 categories: “January” to “December”	BO and BL
*ArrivalDateWeekNumber*	Integer	Week number of the arrival date	BO and BL
*ArrivalDateYear*	Integer	Year of arrival date	BO and BL
*AssignedRoomType*	Categorical	Code for the type of room assigned to the booking. Sometimes the assigned room type differs from the reserved room type due to hotel operation reasons (e.g. overbooking) or by customer request. Code is presented instead of designation for anonymity reasons	BO and BL
*Babies*	Integer	Number of babies	BO and BL
*BookingChanges*	Integer	Number of changes/amendments made to the booking from the moment the booking was entered on the PMS until the moment of check-in or cancellation	BO and BL/Calculated by adding the number of unique iterations that change some of the booking attributes, namely: persons, arrival date, nights, reserved room type or meal
*Children*	Integer	Number of children	BO and BL/Sum of both payable and non-payable children
*Company*	Categorical	ID of the company/entity that made the booking or responsible for paying the booking. ID is presented instead of designation for anonymity reasons	BO and BL.
*Country*	Categorical	Country of origin. Categories are represented in the ISO 3155–3:2013 format [6]	BO, BL and NT
			
*CustomerType*	Categorical	Type of booking, assuming one of four categories:	BO and BL
		Contract - when the booking has an allotment or other type of contract associated to it;	
		Group – when the booking is associated to a group;	
		Transient – when the booking is not part of a group or contract, and is not associated to other transient booking;	
		Transient-party – when the booking is transient, but is associated to at least other transient booking	
*DaysInWaitingList*	Integer	Number of days the booking was in the waiting list before it was confirmed to the customer	BO/Calculated by subtracting the date the booking was confirmed to the customer from the date the booking entered on the PMS
			
*DepositType*	Categorical	Indication on if the customer made a deposit to guarantee the booking. This variable can assume three categories:	BO and TR/Value calculated based on the payments identified for the booking in the transaction (TR) table before the booking׳s arrival or cancellation date.
		No Deposit – no deposit was made;	
			In case no payments were found the value is “No Deposit”.
			If the payment was equal or exceeded the total cost of stay, the value is set as “Non Refund”.
		Non Refund – a deposit was made in the value of the total stay cost;	
			Otherwise the value is set as “Refundable”
		Refundable – a deposit was made with a value under the total cost of stay.	
*DistributionChannel*	Categorical	Booking distribution channel. The term “TA” means “Travel Agents” and “TO” means “Tour Operators”	BO, BL and DC
*IsCanceled*	Categorical	Value indicating if the booking was canceled (1) or not (0)	BO
*IsRepeatedGuest*	Categorical	Value indicating if the booking name was from a repeated guest (1) or not (0)	BO, BL and C/ Variable created by verifying if a profile was associated with the booking customer. If so, and if the customer profile creation date was prior to the creation date for the booking on the PMS database it was assumed the booking was from a repeated guest
*LeadTime*	Integer	Number of days that elapsed between the entering date of the booking into the PMS and the arrival date	BO and BL/ Subtraction of the entering date from the arrival date
*MarketSegment*	Categorical	Market segment designation. In categories, the term “TA” means “Travel Agents” and “TO” means “Tour Operators”	BO, BL and MS
			
*Meal*	Categorical	Type of meal booked. Categories are presented in standard hospitality meal packages:	BO, BL and ML
		Undefined/SC – no meal package;	
		BB – Bed & Breakfast;	
		HB – Half board (breakfast and one other meal – usually dinner);	
		FB – Full board (breakfast, lunch and dinner)	
*PreviousBookingsNotCanceled*	Integer	Number of previous bookings not cancelled by the customer prior to the current booking	BO and BL / In case there was no customer profile associated with the booking, the value is set to 0. Otherwise, the value is the number of bookings with the same customer profile created before the current booking and not canceled.
*PreviousCancellations*	Integer	Number of previous bookings that were cancelled by the customer prior to the current booking	BO and BL/ In case there was no customer profile associated with the booking, the value is set to 0. Otherwise, the value is the number of bookings with the same customer profile created before the current booking and canceled.
*RequiredCardParkingSpaces*	Integer	Number of car parking spaces required by the customer	BO and BL
			
*ReservationStatus*	Categorical	Reservation last status, assuming one of three categories:	BO
		Canceled – booking was canceled by the customer;	
		Check-Out – customer has checked in but already departed;	
		No-Show – customer did not check-in and did inform the hotel of the reason why	
*ReservationStatusDate*	Date	Date at which the last status was set. This variable can be used in conjunction with the *ReservationStatus* to understand when was the booking canceled or when did the customer checked-out of the hotel	BO
*ReservedRoomType*	Categorical	Code of room type reserved. Code is presented instead of designation for anonymity reasons	BO and BL
*StaysInWeekendNights*	Integer	Number of weekend nights (Saturday or Sunday) the guest stayed or booked to stay at the hotel	BO and BL/ Calculated by counting the number of weekend nights from the total number of nights
*StaysInWeekNights*	Integer	Number of week nights (Monday to Friday) the guest stayed or booked to stay at the hotel	BO and BL/Calculated by counting the number of week nights from the total number of nights
*TotalOfSpecialRequests*	Integer	Number of special requests made by the customer (e.g. twin bed or high floor)	BO and BL/Sum of all special requests

a ID is presented instead of designation for anonymity reasons.

The PMS assured no missing data exists in its database tables. However, in some categorical variables like Agent or Company, “NULL” is presented as one of the categories. This should not be considered a missing value, but rather as “not applicable”. For example, if a booking “Agent” is defined as “NULL” it means that the booking did not came from a travel agent.

Summary statistics for both hotels datasets are presented in Table 2, Table 3, Table 4, Table 5, Table 6, Table 7. These statistics were obtained using the ‘skimr’ R package [7].

**Table 2:** H1 dataset summary statistics – Date variables.

**Variable**	**Min**	**Max**	**Median**	**Unique**
*ReservationStatusDate*	2014-11-18	2017-09-14	2016-07-31	913

**Table 3:** H1 dataset summary statistics – Categorical variables.

**Variable**	**Unique**	**Top counts**
*Agent*	186	240: 13 095, NULL: 8 209, 250: 2 869, 241: 1 721
*ArrivalDateMonth*	12	Aug: 4 894, Jul: 4 573, Apr: 3 609, May: 3 559
*AssignedRoomType*	11	A: 17 046, D: 10 339, E: 5 638, C: 2 214
*Company*	236	NULL: 36 952, 223: 784, 281: 138, 154: 133
*Country*	125	PRT: 17 630, GBR: 6 814, ESP: 3 957, IRL: 2 166
*CustomerType*	4	Tra.: 30 209, Tra.-Party: 7 791, Con.: 1 776, Gro.:284
*DepositType*	3	No Dep.: 38 199, Non-Refund.: 1 719, Ref.: 142
*DistributionChannel*	4	TA/TO: 28 295, Dir.: 7 865, Cor.: 3 269, Und.: 1
*IsCanceled*	2	0: 28 938, 1: 11 122
*IsRepeatedGuest*	2	0: 38 282, 1: 1 778
*MarketSegment*	6	Onl.: 17 729, Off.: 7472, Dir.: 6 513, Gro.: 5 836
*Meal*	5	BB: 30 005, HB: 8 046, Und.: 1 169, FB: 754
*ReservationStatus*	3	C.Out: 28 938, Can.: 10 831, No-Show: 291
*ReservedRoomType*	10	A: 23 399, D: 7 433, E: 4 892, G: 1610

**Table 4:** H1 dataset summary statistics – Integer and numeric variables.

**Variable**	**Mean**	**SD**	**P0**	**P25**	**Median**	**P75**	**P100**
*ADR*	94.95	61.44	-6.38	50	75	125	508
*Adults*	1.87	0.7	0	2	2	2	55
*ArrivalDateOfMonth*	15.82	8.88	1	8	16	24	31
*ArrivalDateWeekNumber*	27.14	14.01	1	16	28	38	53
*ArrivalDateYear*	2016.12	0.72	2015	2016	2016	2017	2017
*Babies*	0.014	0.12	0	0	0	0	2
*BookingChanges*	0.29	0.73	0	0	0	0	17
*Children*	0.13	0.45	0	0	0	0	10
*DaysInWaitingList*	0.53	7.43	0	0	0	0	185
*LeadTime*	92.68	97.29	0	10	57	155	737
*PreviousBookingsNotCanceled*	0.15	1	0	0	0	0	30
*PreviousCancellations*	0.1	1.34	0	0	0	0	26
*RequiredCarParkingSpaces*	0.14	0.35	0	0	0	0	8
*StaysInWeekendNights*	1.19	1.15	0	0	1	2	19
*StaysInWeekNights*	3.13	2.46	0	1	3	5	50
*TotalOfSpecialRequests*	0.62	0.81	0	0	0	1	5

**Table 5:** H2 dataset summary statistics – Date variables.

**Variable**	**Min**	**Max**	**Median**	**Unique**
*ReservationStatusDate*	2014-10-17	2017-09-07	2016-08-10	864

**Table 6:** H2 dataset summary statistics – Categorical variables.

**Variable**	**Unique**	**Top counts**
Agent	224	9: 31 955, NULL: 8 131, 1: 7 137, 14: 3 640
*ArrivalDateMonth*	12	Aug: 8 983, May: 8 232, Jul: 8 088, Jun: 7 894
*AssignedRoomType*	9	A: 57 007, D: 14 983, E: 2 168, F: 2 018
*Company*	208	NULL: 75 641, 40: 924, 67: 267, 45: 250
*Country*	166	PRT: 30 960, FRA: 8 804, DEU: 6 084, GBR: 5315
*CustomerType*	4	Tra.:59 404, Tra.-P.: 17 333, Con.: 2 300, Gro.:293
*DepositType*	3	No Dep.: 66 442, Non-Refund.: 12 868, Ref.: 20
*DistributionChannel*	5	TA/TO: 68 945, Dir.: 6 780, Cor.: 3 408, GDS: 193
*IsCanceled*	2	0: 46 228, 1: 33 102
*IsRepeatedGuest*	2	0: 77 298, 1: 2 032
*MarketSegment*	8	Onl.: 38 748, Off.: 16 747, Gro.: 13 975, Dir.: 6 093
*Meal*	4	BB: 62 305, SC: 10 564, HB: 6 417, FB: 44
*ReservationStatus*	3	C.Out: 46 228, Can.: 32 186, No-Show: 916
*ReservedRoomType*	8	A: 62 595, D: 11768, F: 1 791, E: 1 553

**Table 7:** H2 dataset summary statistics – Integer and numeric variables.

**Variable**	**Mean**	**SD**	**P0**	**P25**	**Median**	**P75**	**P100**
*ADR*	105.3	43.6	0	79.2	99.9	126	5400
*Adults*	1.85	0.51	0	2	2	2	4
*ArrivalDateOfMonth*	15.79	8.73	1	8	16	23	31
*ArrivalDateWeekNumber*	27.18	13.4	1	17	27	38	53
*ArrivalDateYear*	2016.17	0.7	2015	2016	2016	2017	2017
*Babies*	0.0049	0.084	0	0	0	0	10
*BookingChanges*	0.19	0.61	0	0	0	0	21
*Children*	0.091	0.37	0	0	0	0	3
*DaysInWaitingList*	3.23	20.87	0	0	0	0	391
*LeadTime*	109.74	110.95	0	23	74	163	629
*PreviousBookingsNotCanceled*	0.13	1.69	0	0	0	0	72
*PreviousCancellations*	0.08	0.42	0	0	0	0	32
*RequiredCarParkingSpaces*	0.024	0.15	0	0	0	0	3
*StaysInWeekendNights*	0.8	0.89	0	0	1	2	16
*StaysInWeekNights*	2.18	1.46	0	1	2	3	41
*TotalOfSpecialRequests*	0.55	0.78	0	0	0	1	5

**Fig. 2:**
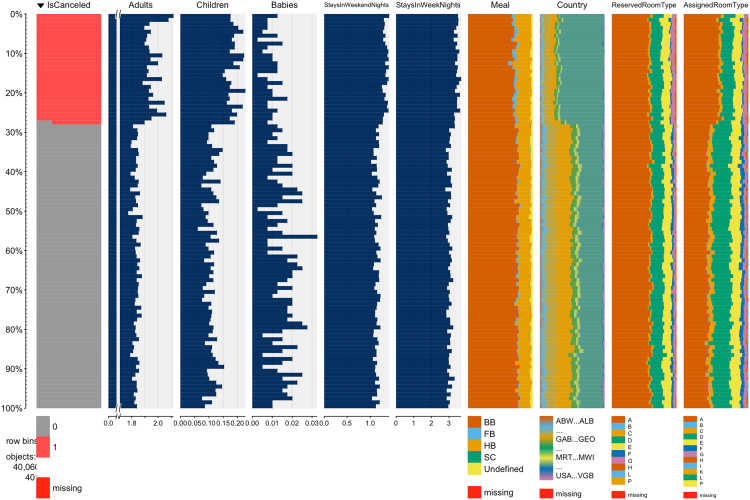
H1 dataset partial visualization of all observations.

**Fig. 3:**
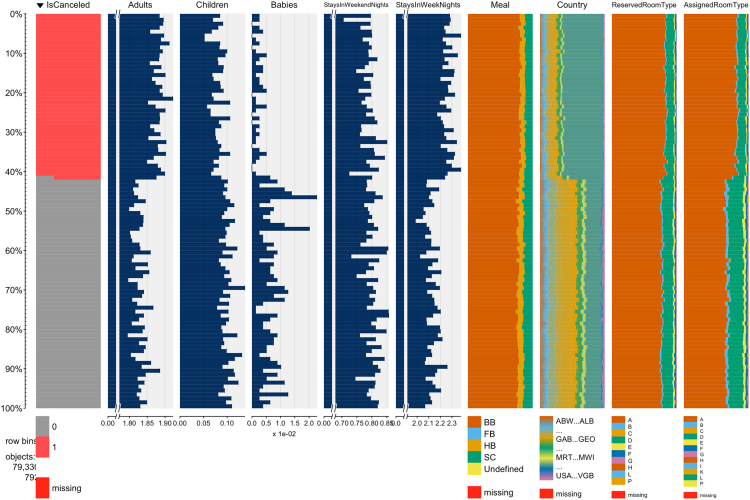
H2 dataset partial visualization of all observations.

A word of caution is due for those not so familiar with hotel operations. In hotel industry it is quite common for customers to change their booking׳s attributes, like the number of persons, staying duration, or room type preferences, either at the time of their check-in or during their stay. It is also common for hotels not to know the correct nationality of the customer until the moment of check-in. Therefore, even though the capture of data took considered a timespan prior to arrival date, it is understandable that the distribution of some variables differ between non canceled and canceled bookings. Consequently, the use of these datasets may require this difference in distribution to be taken into account. This difference can be seen in the table plots of Fig. 2 and Fig. 3. Table plots are a powerful visualization method and were produced with the tabplot R package [8] that allow for the exploration and analysis of large multivariate datasets. In table plots each column represents a variable and each row a bin with a pre-defined number of observations. In these two figures, each bin contains 100 observations. The bars in each variable show the mean value for numeric variables or the frequency of each level for categorical variables. Analyzing these figures it is possible to verify that, for both of the hotels, the distribution of variables like *Adults*, *Children*, *StaysInWeekendNights*, *StaysInWeekNights*, *Meal*, *Country* and *AssignedRoomType* is clearly different between non-canceled and canceled bookings.
